# Mandibular Implants: A Metamaterial‐Based Approach to Reducing Stress Shielding

**DOI:** 10.1002/adhm.202500405

**Published:** 2025-04-04

**Authors:** Jorn‐Ids Heins, Bram B.J. Merema, Joep Kraeima, Max J.H. Witjes, Anastasiia O. Krushynska

**Affiliations:** ^1^ Department of Oral and Maxillofacial Surgery University of Groningen University Medical Center Groningen Groningen 9713 GZ The Netherlands; ^2^ 3D Lab University Medical Center Groningen University of Groningen Groningen 9713 GZ The Netherlands; ^3^ Engineering and Technology Institute Groningen University of Groningen Groningen 9747AG The Netherlands

**Keywords:** implant, mandible, Metamaterial, photoelasticity, porous

## Abstract

Biomechanical complications, such as stress shielding, bone resorption, and reconstruction failure, are prevalently associated with solid titanium mandible reconstruction plates. This study evaluates the potential of metamaterial designs with porous gyroid microarchitectures, to enhance biomechanical stimulation and mitigate these complications. A novel metamaterial reconstruction plate is compared with solid titanium plates, both patient‐specifically designed and fabricated from Ti6Al4 V alloy. Stress shielding is assessed through photoelasticity experiments and validated with finite element analysis (FEA). Transparent mandible models are loaded incrementally (0–1000 N) to analyze stress distributions in the implants, screws, and mandible segments. The metamaterial plate reduces stress concentrations in the distant mandibular regions from the defect, while increasing stress around the screws near the defect, favoring local mechanical stimulation. FEA confirms improved load distribution (p = 0.003). However, the metamaterial plate exhibited a lower load‐bearing capacity, failing at 775 N, while the solid plate withstood 1800 N without failure. Yet, the metamaterial design effectively reduced stress shielding, thereby enhancing biomechanical function near critical mandibular regions. Hence, despite their reduced load‐bearing capacity, they can, potentially, preserve bone integrity and prevent implant failure that should be validated in future (pre‐)clinical studies.

## Graphical abstract

Metamaterial mandibular implants offer a novel approach to reducing stress shielding and enhancing biomechanical stimulation. This study compares a gyroid‐based metamaterial plate to a solid titanium plate using photoelasticity and finite element analysis. Results show improved stress distribution and localized mechanical stimulation, highlighting the potential of metamaterial implants for optimizing load transfer and promoting bone integrity.

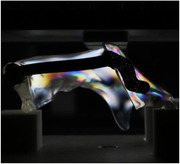

## 1 Introduction

Stress shielding is a persistent problem for femoral,^[^ ^1^ ^]^ spinal,^[^ ^2^ ^]^ mandibular,^[^ ^3^ ^]^ dental,^[^ ^4^ ^]^ and other load‐bearing implants caused by a mismatch in mechanical behaviors at implant‐bone interfaces.^[^ ^5^ ^]^ Stress shielding leads to a redistribution of the load transfer to the bones, which decreases implant stability, induces aseptic loosening and wear, and initiates bone resorption in a relatively short time.^[^ ^6^ ^]^ As a consequence, patients experience excessive bone loss and pain, and ultimately require revision surgery due to implant failure.^[^ ^7^ ^]^

Many researchers, with the aim to reduce stress shielding, have proposed alternative constituent materials,^[^ ^2^, ^8^ ^]^ porous designs,^[^ ^9^ ^]^ interfacial coatings and layers,^[^ ^10^ ^]^ or optimized implant topology.^[^ ^11^ ^]^ These solutions can achieve a more balanced load distribution but, at the same time, may deliver inconsistent results, raise implementation challenges, or biological and biomechanical concerns.^[^ ^12^ ^]^ For instance, thermoplastic polymers used as substitutes for metal alloys in implants suffer from poor biocompatibility,^[^ ^13^ ^]^ toxicity,^[^ ^14^ ^]^ osseointegration issues,^[^ ^10b^ ^]^ and high stresses, consequently inducing periprosthetic fractures,^[^ ^10b^ ^]^ fatigue and safety threats.^[^ ^8^, ^11^ ^]^

Recently, a promising avenue emerged to mitigate stress shielding using meta‐implants –, that is, comprised of metamaterials.^[^ ^15^ ^]^ Metamaterials are architected media that can be made from any constituent material, and have a fine‐tuned micro‐structure governing their mechanical response. The distinct feature of metamaterials is that their mechanical properties can be manipulated into broad (stiffness, strength, anisotropy, etc.) and even unexpected (rotate when compressed, expand when pulled, become fluid‐like solids, undergo shape‐morphing, etc.) ranges by adjusting the geometric parameters, e.g., the size, shape, and composition of the micro‐structural elementary building blocks.^[^ ^15^, ^16^ ^]^

Many studies have proposed meta‐implant designs, including topology optimized,^[^ ^17^ ^]^ hybrid,^[^ ^18^ ^]^ gradient,^[^ ^19^ ^]^ lattice‐type,^[^ ^20^ ^]^ auxetic,^[^ ^15a^ ^]^ and bone‐mimicking micro‐structures,^[^ ^21^ ^]^ as well as truly smart multi‐functional self‐healing,^[^ ^22^ ^]^ self‐sensing,^[^ ^23^ ^]^ and self‐deployable^[^ ^24^ ^]^ meta‐implants. Due to the geometric complexity and manufacturing constraints, meta‐implant designs are often developed by numerical studies replicating the presence of bones by equivalent loadings, and their micro‐structure is optimized whilst ignoring the presence of fixation screws.^[^ ^3^, ^25^ ^]^ Screws and their mechanical interactions with the bones can, however, significantly modify the stress fields within an implant and the bones, which can lead to sub‐optimal performance and even enhanced stress shielding.^[^ ^11^, ^19^, ^26^ ^]^

We developed an experimental‐numerical framework to estimate stress shielding induced by load‐bearing metamaterial implants in preclinical conditions. The experimental analysis was conducted with a previously validated optical non‐contact photoelasticity technique for solid implants,^[^ ^27^ ^]^ and the numerical simulations were performed on full‐scale finite‐element implant‐bone‐screw models. We studied the stresses produced by three mandibular reconstruction plates – conventional manually bent, patient‐specific solid, and patient‐specific metamaterial designs – fixed to transparent plastic bone analogs by self‐tapping screws and subjected to step‐varying loading conditions. We hypothesized that the stress distribution of the metamaterial implant will be more favorable within the mandible, thus enhancing the mechanical stimuli in typical stress‐shielding areas.

## 2 Design and Analysis Steps

Conventional or patient‐specific reconstruction plates are used to bridge a continuity defect via mandibular reconstruction surgery when the patient's condition does not allow for a free vascularized flap, or the patient refuses surgery on the donor site. Such plates are typically made from a medical‐grade titanium alloy, due to its high strength and proven biocompatibility.^[^ ^28^ ^]^

### 2.1 Reconstruction Plate Design

**Figure 1:**
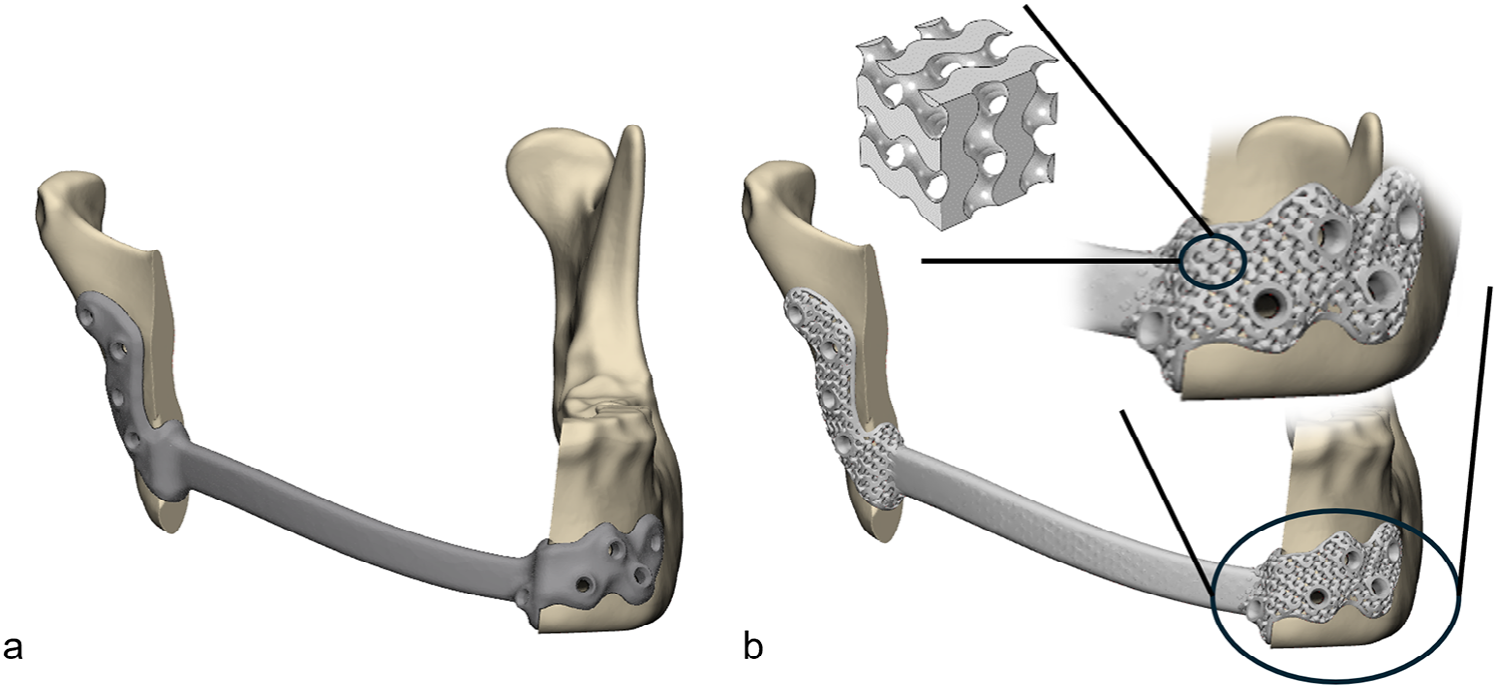
Patient‐specific designs of mandibular reconstruction plates visualized in 3‐Matic: a) the solid reconstruction plate (reference) and b) the metamaterial reconstruction plate with the zoomed‐in flange illustrating the metamaterial micro‐structure and thin solid edges around the flange and the screw holes. The smallest inset illustrates a block of 2 by 2 by 2 gyroid unit cells.

We designed two patient‐specific mandible reconstruction plates using 3‐Matic Medical 18.0 (Materialise, Leuven, Belgium). The bone segmentation and virtual surgical planning were performed using Mimics Medical 26.0 (Materialise, Leuven, Belgium). One of the plates – a solid patient‐specific reconstruction plate (**Figure** **1**a), designed according to the Univeristy Medical Center Groningen (UMCG) standard,^[^ ^11f^ ^]^ was milled from medical grade 23 ELI titanium alloy (Witec Medical B.V., Stadskanaal, the Netherlands). The second plate, with the same gross shape and volume, was designed with metamaterial parts (flanges) at the implant ends (Figure 1b).

The metamaterial parts consisted of periodic unit cells formed by gyroids (Figure 1b) which belong to the Triply Periodic Minimal Surfaces (TPMS) family. We opted for this design due to the continuous smooth surfaces in the gyroid micro‐structure, hence ensuring a uniform distribution of stress and a reduction in stress concentrations.^[^ ^29^ ^]^ Moreover, gyroids can be easily manufactured by means of medically certified additive manufacturing techniques and have been widely used in the design of implants.^[^ ^30^ ^]^

The gyroid metamaterials were created in nTop (nTop, New York, USA); the geometric details are provided in the next section. Since replacing the solid parts with a gyroid metamaterial increases the volume, we used the metamaterial only in the flanges to keep the implant bridge as small as possible to reduce the risk of exposure due to soft tissue recession. Next, we introduced thin solid edges around the thinnest metamaterial parts of the implant to enable the placement of a support during manufacturing, and around the screw holes to enable proper fixation. We hypothesized that by reducing the effective stiffness of the flanges locally, we would modify the load transfer conditions and thus increase mechanical stimulation of the mandible near the implant‐bone interfaces, which should reduce stress shielding.

The metamaterial patient‐specific reconstruction plate was 3D printed from medical grade 23 titanium alloy (Mobius 3D Technologies B.V., Nieuw‐Vennep, the Netherlands) under Deutsche Institut für Normung (DIN) and the International Organization for Standardization (ISO) 2768‐T1 standards (±0.1 mm overall tolerance, screw holes ±0.05 mm tolerance) and then subjected to heat treatment in a 0.001 mBar vacuum at 840 °C for 150 min. Both plates were compatible with 2.3 mm ThreadLock locking screws (KLS Matin, Tuttlingen, Germany). Finally, we also considered a (third) conventional titanium osteosynthesis mandible reconstruction plate (KLS Martin, Tuttlingen, Germany) bent to a desired shape by a trained and experienced surgical assistant.

### 2.2 Metamaterial Design

Despite endless theoretical design possibilities for metamaterials, the design of a metamaterial reconstruction plate requires maintaining the physical feasibility and producibility of the geometric features of the microstructure, which also depend on a constituent material and manufacturing process. Hence, to ensure clinical applicability, the metamaterial implant was produced from Ti6Al4 V medical grade titanium by laser powder bed fusion additive manufacturing – the certified production technique for state‐of‐the‐art implants. This process restricts the minimal wall thickness to 0.2 mm, the minimal pore size to 0.2 mm, the maximum 90‐degree overhang to 0.5 mm, and the minimum unsupported overhang angle to 45 degrees.

Yet, to avoid size effects and to achieve the theoretical estimates of the effective mechanical properties of the metamaterial, the number of unit cells in the microstructure should be maximized.^[^ ^31^ ^]^ These contradictory requirements, and a clinical limitation to keep the implant sizes as compact as possible, resulted in the need to maximize the number of unit cells by reducing the unit cell size. To realize the mechanical properties of cortical bone, i.e., E_b_ = 14.8 GPa,^[^ ^32^ ^]^ while accounting for the difference in volume between the resected bone and the gross‐geometry of the implant and manufacturability, we chose a gyroids material with a relative density of 50% and a unit cell size of 1.75 mm that resulted in an effective metamaterial with a Young's modulus of E_m_ = 18 GPa.^[^ ^33^ ^]^

### 2.3 Photoelasticity Testing

To experimentally study stress shielding, we used a photoelasticity testing procedure that visualizes isochromatic fringes (stress distribution) within a transparent material. It involves using polarized light to observe stress‐induced birefringence patterns.^[^ ^34^ ^]^

The patient's mandible was segmented based on Computed Tomography (CT) images, exported as an stereolithography (STL) file, and additively manufactured using a Stereolithography Apparatus (SLA) Formlabs 3D printer from Clear V4 resin (Formlabs, Berlin, Germany). Despite the mechanical properties of the resin differing from those of the mandible, its geometrical identity enables correct visualization of the stress distribution areas induced by the three reconstruction plates, thus justifying our qualitative and comparative study of the stress fields.

To ensure a high‐level of transparency of the resin replicas of the mandible, the 3D‐printed parts were manually polished to a desired high‐gloss finish. Next, they were connected to the designed reconstruction plates by self‐tapping 2.3 mm ThreadLock screws, which were placed in the pre‐designed 1.9 mm diameter holes corresponding to the drill used during surgeries (KLS Martin, Tuttlingen, Germany).

**Figure 2:**
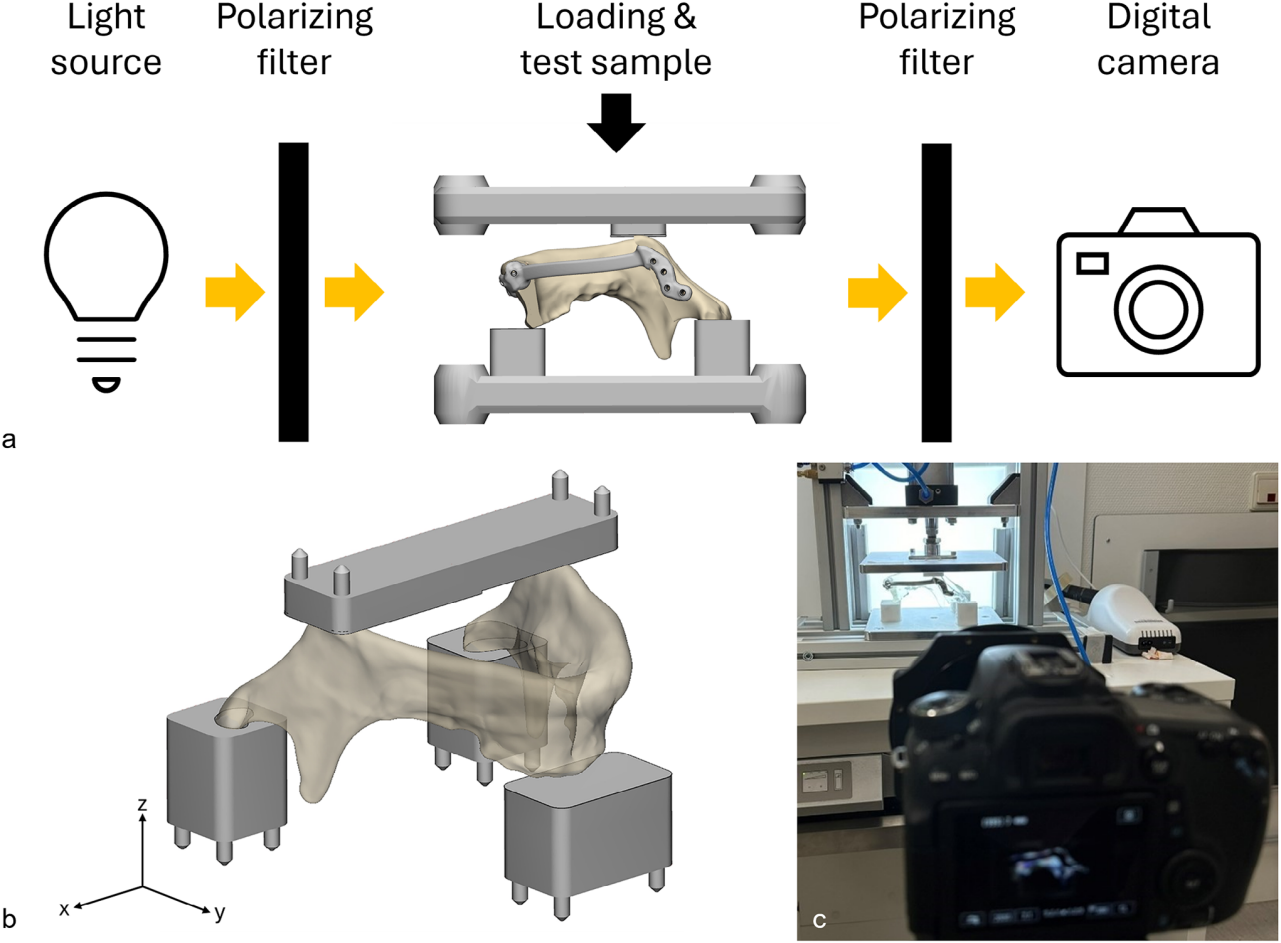
a) Schematics of the photoelasticity testing setup. The light from the source on the left is polarized by the first polarizing filter and is transmitted through the test sample. The digital camera on the right captures the images through another polarizing filter in front of it. b) Inserts for the compression testing setup with the mandible placed upside down, and c) photograph of the test setup showing the light source with polarizing filter in the background, the compression bench with a mandible and solid reconstruction plate in the middle, and the camera with the second polarizing filter in the foreground.

To induce stress in the assembled parts, we developed a uniform compression setup consisting of a compression bench with two aluminum plates which could be fitted with inserts (3D printed from polyamide 12 (PA12) by Oceanz BV, Ede, the Netherlands), as shown in **Figure** **2**b. The inserts were designed specifically for the test samples such that the condyles of the mandible could rest freely on them, ensuring the desired fixed position in both the horizontal (x‐y plane) and the negative vertical (z) direction. On the other hand, the inserts for the mandibular angles were flat and had no recess, which allowed for translation of the mandibular angles in the horizontal plane upon loading. Although severely oversimplified,^[^ ^12a^ ^]^ this setup is in‐line with generally accepted compression testing setups for mandibles described elsewhere.^[^ ^3^, ^35^ ^]^ The coronoid processes of the mandible were also not constrained, allowing the mandible to flex freely under loading.

The photoelasticity testing procedure is schematically shown in Figure 2a. A polarizing filter was placed in front of an 1800 lm Light Emitting Diode (LED) panel (30 cm × 30 cm, 6000 K), both of which were positioned behind the compression bench. A Canon EOS 7D camera, with a 50 mm lens and a 105 mm polarizing filter, was placed on a tripod in front of the compression bench. The ISO setting on the camera was 3200, the shutter time was 1/2000 s, the aperture was 7.1, and the focus was manual. The compression bench and the camera were fixed. A photo of the described testing setup is given in Figure 2c.

### 2.4 Experimental Steps and Image Processing

The test samples were loaded step‐wise from 0 to 1000 N with an increment of 100 N. This range exceeds the maximum expected physiological loads created during mastication.^[^ ^12^, ^36^ ^]^ The tests were repeated for four different camera angles: the front view, right‐side view, left‐side view and rear view. These views were obtained by rearranging each test specimen and inserts in the compression bench along a pre‐determined direction. During the tests, the room was completely dark to prevent any light interference, and the room temperature was preserved at 20 °C.

The original images were used to inspect and compare the induced stress fields visually. Additionally, copies of the images were post processed using color mapping by means of a Python script, with the aim to discretize the continuous color images into distinct color segments. The script was used to map the real captured colors into six colors in the standard Blue, Green, and Red (BGR) format. Specifically, the color thresholds were set as follows: red [0, 0, 100]‐[100, 100, 255], green [0, 100, 0]‐[100, 255, 100], blue [100, 0, 0]‐[255, 100, 100], yellow [0, 100, 100]‐ [100, 255, 255], cyan [100, 100, 0]‐[255, 255, 100], and magenta [100, 0, 100]‐[255, 100, 255]. Based on these thresholds, binary masks were created that identified pixels within the specified color ranges of an image. Each pixel falling within one of the thresholds was returned as the corresponding color, using the BGR codes, i.e., red [0, 0, 255], green [0, 255, 0], blue [255, 0, 0], yellow [0, 255, 255], cyan [255, 255, 0], and magenta [255, 0, 255]. The number of isochromatic fringes were counted for each of the colored areas created in this way, providing the data for the quantitative analysis.

### 2.5 Finite Element Analysis

To analyze the stresses numerically, and to compare them with the isochromatic fringe patterns from the photoelasticity tests, we performed two sets of finite‐element studies using Abaqus 2024 (Simulia, Providence, Rhode Island, USA) in the static linear elastic regime. In the first set, we considered the Clear V4 Resin material in the mandible segments, whereas in the second one, we considered those parts modelled with cortical bone properties (**Table** **1**). The latter initially entailed hollowing the mandible segments such that only a 2 mm shell remained which resembled cortical bone.^[^ ^32^, ^37^ ^]^

**Table 1:** Mechanical and material properties used in the finite‐element studies.

Material	Parts	Young's modulus	Poisson ratio	Reference
Titanium alloy	Reconstruction plates and screws	114000 [MPa]	0.3 [‐]	Material data sheet Mobius 3D technologies and Witec Medical B.V.
Clear V4 Resin	Mandible segments	2200 [MPa]	0.3 [‐]	Datasheet Formlabs
Polyamide 12	Inserts	1650 [MPa]	0.3 [‐]	Datasheet Oceanz
Cortical bone	Mandible segments	14800 [MPa]	0.3 [‐]	[32]

We specified the general contact interaction on all surface pairs as displaying frictionless tangential behavior and hard‐contact normal behavior, with separation occurring after contact. The screw surfaces were tied to the corresponding cylindrical holes in the mandible segments and reconstruction plate. The inserts were fixed in all directions. Vertical loads on both the left and right mandibular angle of the mandible segments complied with the loading direction in the photoelasticity tests. The loads were specified by their amplitudes, defined as a 10‐step function from 0 to 1000 N, with an increment of 100 N, also in agreement with the tests.

The mandible segments, inserts, screws, connections, and reconstruction plate were meshed with a tet4 volume finite‐element mesh using 3‐Matic Research 18.0 (Materialise, Leuven, Belgium).

## 3 Results

### 3.1 Photoelasticity Tests

**Figure 3:**
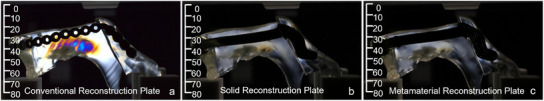
Original photos of unloaded samples with size annotation in mm. a) the manually contoured conventional implant shows substantial stress concentrations within the whole interior of the mandibular segments, probably induced by the manual bending, b) the solid patient‐specific implant shows close‐to‐zero initial stresses, c) the metamaterial patient‐specific implant shows close‐to‐zero initial stresses.

Below are the original photographs of the unloaded test samples (**Figure** **3**). As can be seen, the manually bent plate induced considerable stresses in the mandibular parts, even without any load, while the solid and metamaterial reconstruction plates revealed a negligible initial stress level.

**Figure 4:**
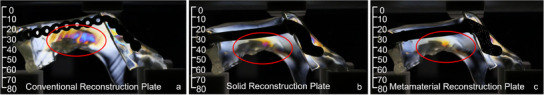
Original photos of the fringe patterns under 300 N loading with size annotation in mm. a) manually‐bent conventional, b) solid patient‐specific, and c) metamaterial patient‐specific reconstruction plates. The conventional plate clearly induced the highest stress in the mandible, with significant concentrations in the left mandible segment. The solid implant also generated more stress compared to the metamaterial implant.

The selected images from the tests depicted in **Figure** **4** indicate the most different isochromatic fringe patterns induced by loading the plates with 300 N. It can be seen that the mandible reconstructed by the manually bent plate experienced significantly higher stresses (light blue in the encircled area) than those in the mandible segments connected by the solid and metamaterial reconstruction plates. Similarly, the solid reconstruction plate introduced slightly more stress in the left mandible segment compared to that by the metamaterial reconstruction plate. The high (initial) stresses induced by the manually bent plate complicated the analysis of the stress fields under higher loadings. Therefore, we then focused on the patient‐specific solid and metamaterial reconstruction plate tests.

**Figure 5:**
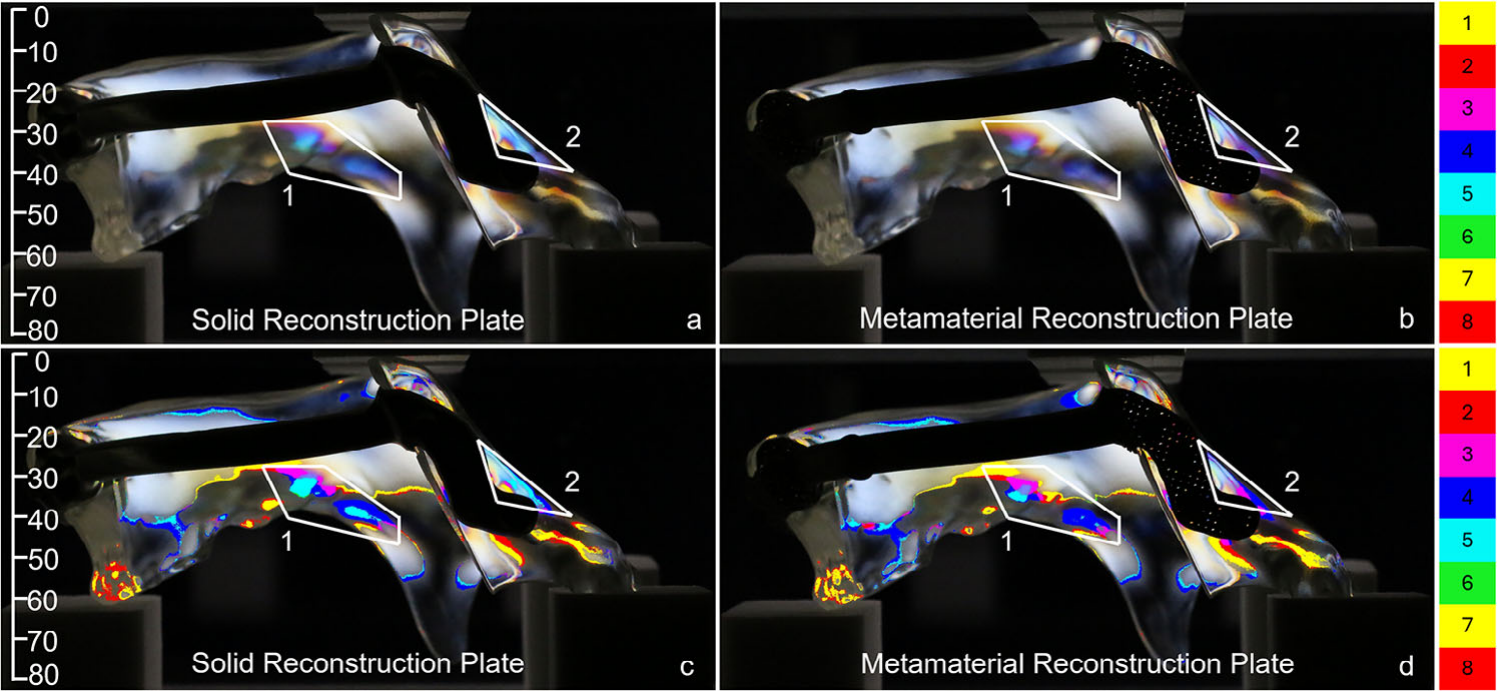
Right view with size annotation in mm. Original a,b) and post‐processed c,d) photos of the fringe patterns under 500 N loading induced by the (a, c) solid and (b, d) metamaterial reconstruction plates. The solid plate induced higher stresses in the left mandible segment (area 1) and in the right mandible segment near the screws (area 2). The post‐processed photos reveal an isochromatic fringe count of six in area 1 and seven in area 2 for the solid plate, and an isochromatic fringe count of five in areas 1 and 2 for the metamaterial plate.

**Figure** **5** shows the original and post‐processed photos of the right view of the solid and metamaterial reconstruction plates loaded with 500 N. The highlighted regions in the mandible segments, namely the molar region of the left mandible segment (area 1) and the ramus region of the right mandible segment (area 2), connected by the solid plate indicate higher stresses compared to those with the metamaterial plate. Specifically, the blue color in area 1 of Figure 5a,c is significantly brighter than that in area 1 of Figure 5b,d, as also confirmed by the discretized isochromatic fringe count of six for the solid reconstruction plate, and five for the metamaterial reconstruction plate. Regarding the ramus (area 2), the screws placed farthest away from the defect introduced more stress in the right mandible segment assembled with a solid reconstruction plate, compared to the metamaterial reconstruction plate. This is underscored by the isochromatic fringe counts of seven and five for these plates, respectively.

**Figure 6:**
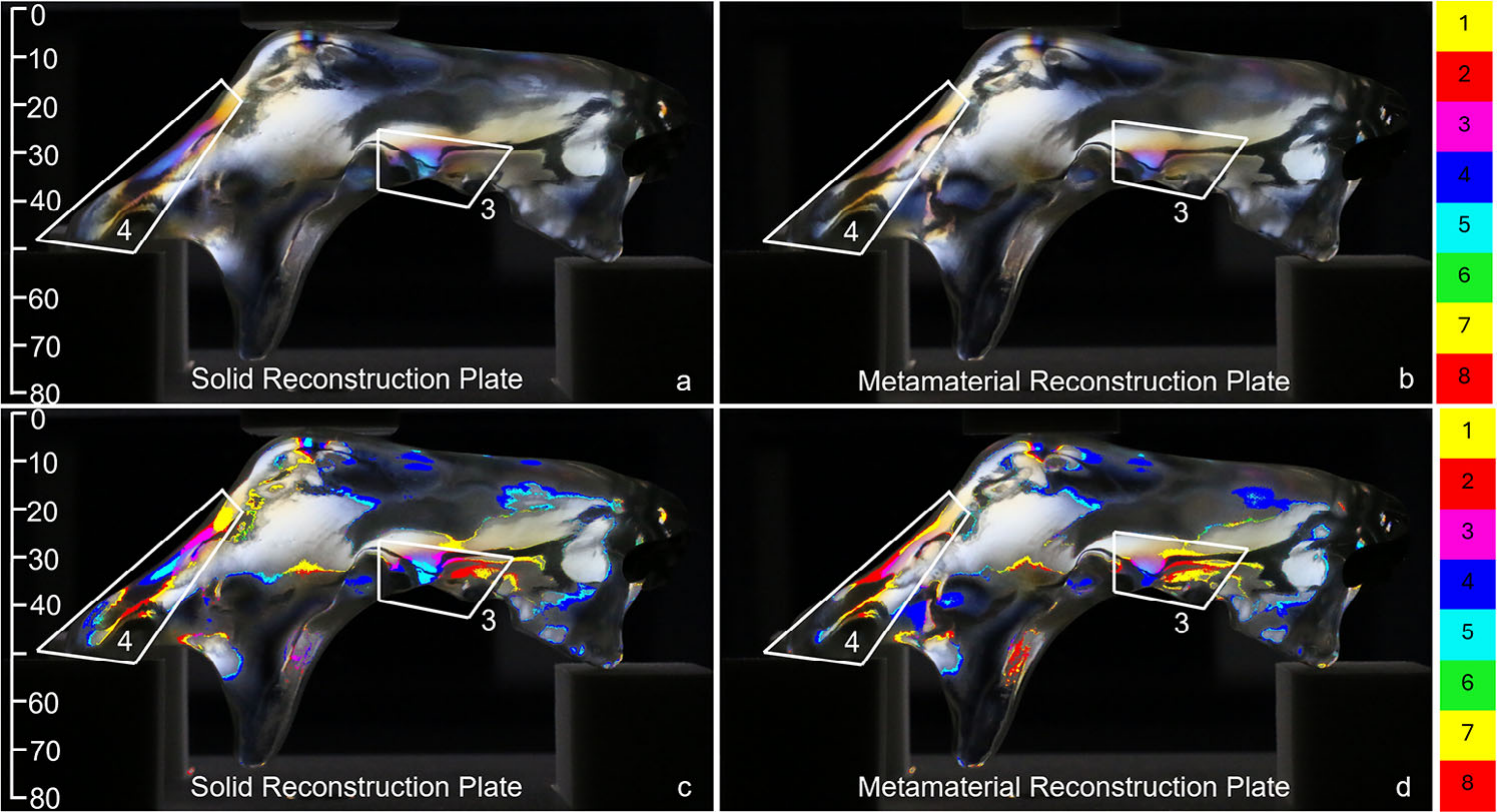
Left view with size annotation in mm. Original a,b) and post‐processed c,d) photos of the fringe patterns under 500 N loading induced by the (a, c) solid and (b, d) metamaterial reconstruction plates. Similar to the right view in Figure 5, the solid plate generated higher stresses in the left molar region (area 3) and the left ramus region (area 4) compared to the metamaterial implant. The post‐processed photos reveal an isochromatic fringe count of five in areas 3 and 4 for the solid plate, and an isochromatic fringe count of four in areas 3, and a count of three in area 4 of the metamaterial plate.

The left views of the samples under the same loading of 500 N are given in **Figure** **6**. Here, the left molar region (area 3) demonstrated larger stresses induced by the solid reconstruction plate compared to the metamaterial reconstruction plate. The corresponding isochromatic fringe counts were five and four, respectively. A similar trend was observed in area 4, corresponding to the left ramus region, where the isochromatic fringe count equaled to five for the solid reconstruction plate and to three for the metamaterial reconstruction plate.

**Figure 7:**
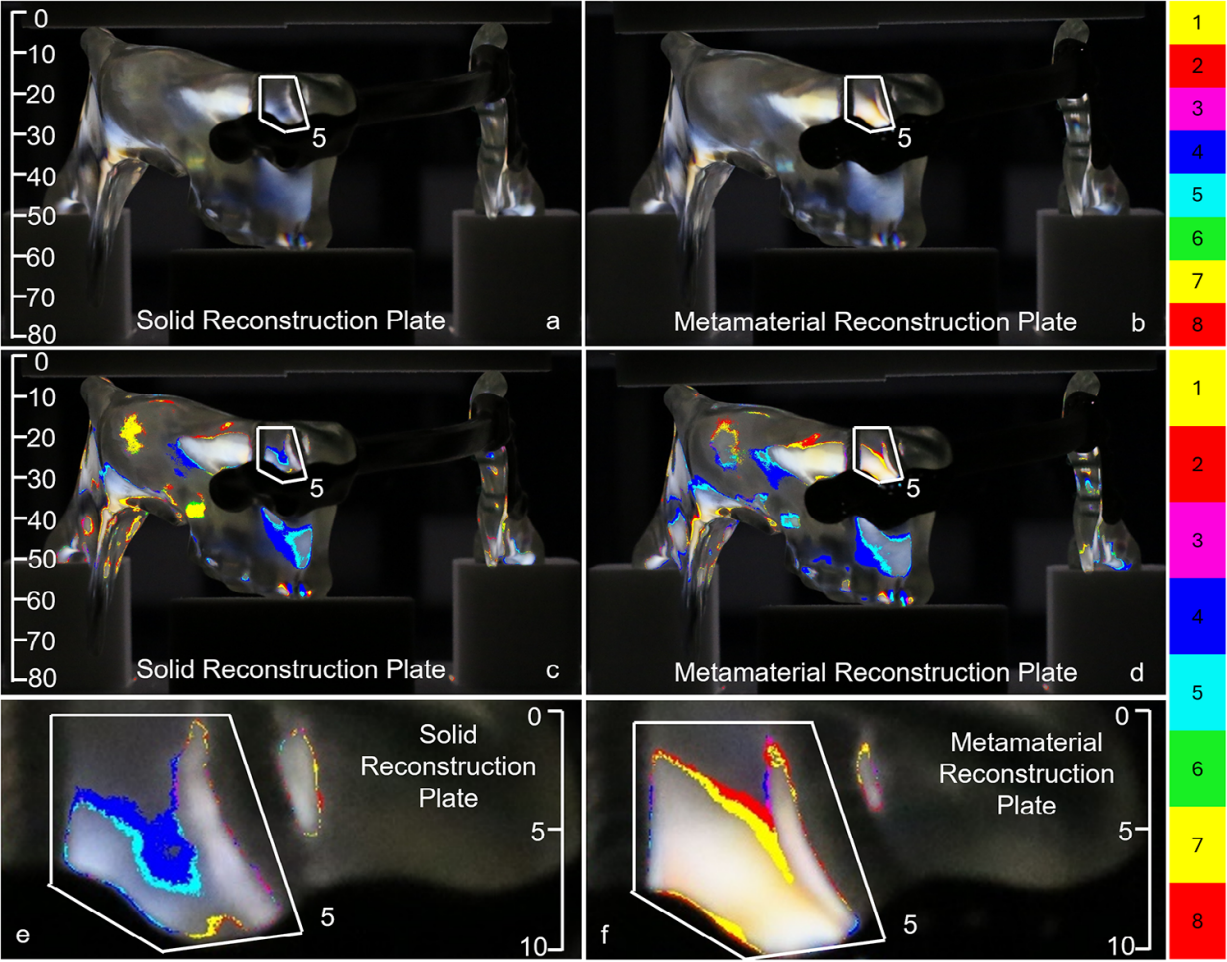
Front view with size annotation in mm. Original a,b) and post‐processed c‐f) photos of the fringe patterns under 500 N loading induced by the (a, c, e) solid and (b, d, f) metamaterial reconstruction plates. In contrast to the right and left views in Figures 5 and 6, the metamaterial plate generated higher stresses in the highlighted area 5 than the solid plate. Specifically, an isochromatic fringe count of five was observed in area 5 of the sold implant, whereas the metamaterial plate had a count of eight. The area close to the defect in the metamaterial test sample (f) is brighter compared to the solid test sample (e).

**Figure 8:**
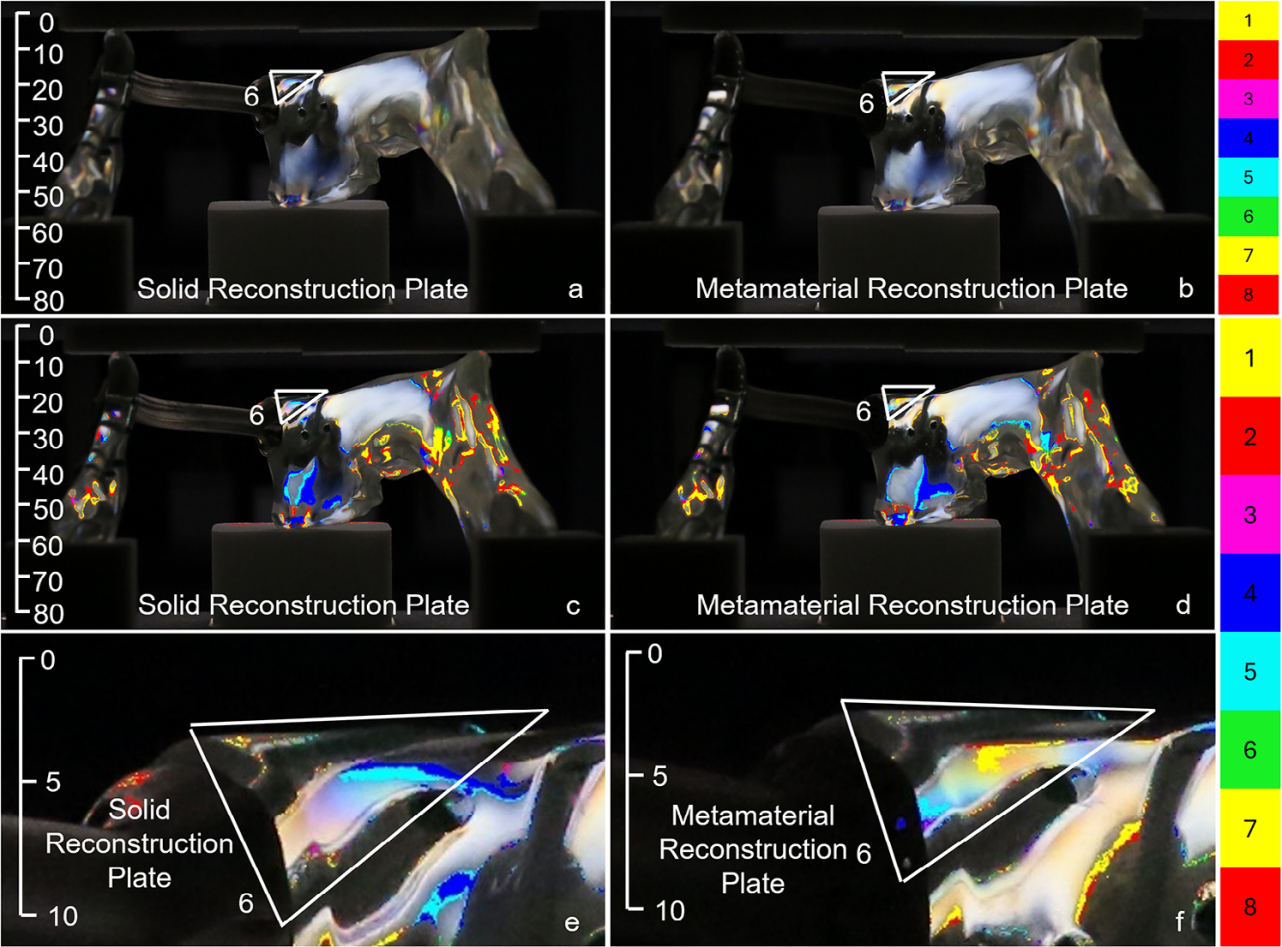
Rear view with size annotation in mm. Original a,b) and post‐processed c‐f) photos of the fringe patterns under 500 N loading induced by the (a, c, e) solid and (b, d, f) metamaterial reconstruction plates. Similar to the front view in Figure 7, the metamaterial plate generated higher stresses in the highlighted area 6 than that induced by the solid plate. Notably, an isochromatic fringe count of five was observed in area 6 of the sold implant, while the metamaterial plate had a count of seven. The area close to the defect in the metamaterial test sample (f) is brighter compared to the solid test sample (e), indicating higher stresses.

A similar pattern was observed on inspecting the front view of the solid and metamaterial reconstruction plates, as shown in **Figure** **7**. Note that the screws located close to the defect introduced more stresses in the left mandible segment connected by the metamaterial plate, see, e.g., the isochromatic fringe count of five in area 5 of the solid reconstruction plate, and of eight for the metamaterial plate. This was corroborated on inspecting the rear view, as depicted in **Figure** **8**, with an isochromatic fringe count of 5 for area 6 of the solid reconstruction plate and a count of 7 for the metamaterial reconstruction plate.

At the end of the tests, the manually bent conventional plate failed on breaking under a load of ≈375 N. The metamaterial reconstruction plate also failed, but under more than twice the load, ≈775 N. In the latter case, the failure was preceded by snapping sounds under loads of 500–600 N, indicating that the thin micro‐structural elements had started to fail in a successive manner, and continued on increasing the load from 600 to 700 N. The solid mandible reconstruction plate survived a load of 1800 N, and did not fail. The failure of the metamaterial plate under lower loadings than those for the solid plate was expected because we had intentionally reduced the plate stiffness by introducing thin microstructural features.

### 3.2 Finite Element Analysis

**Figure 9:**
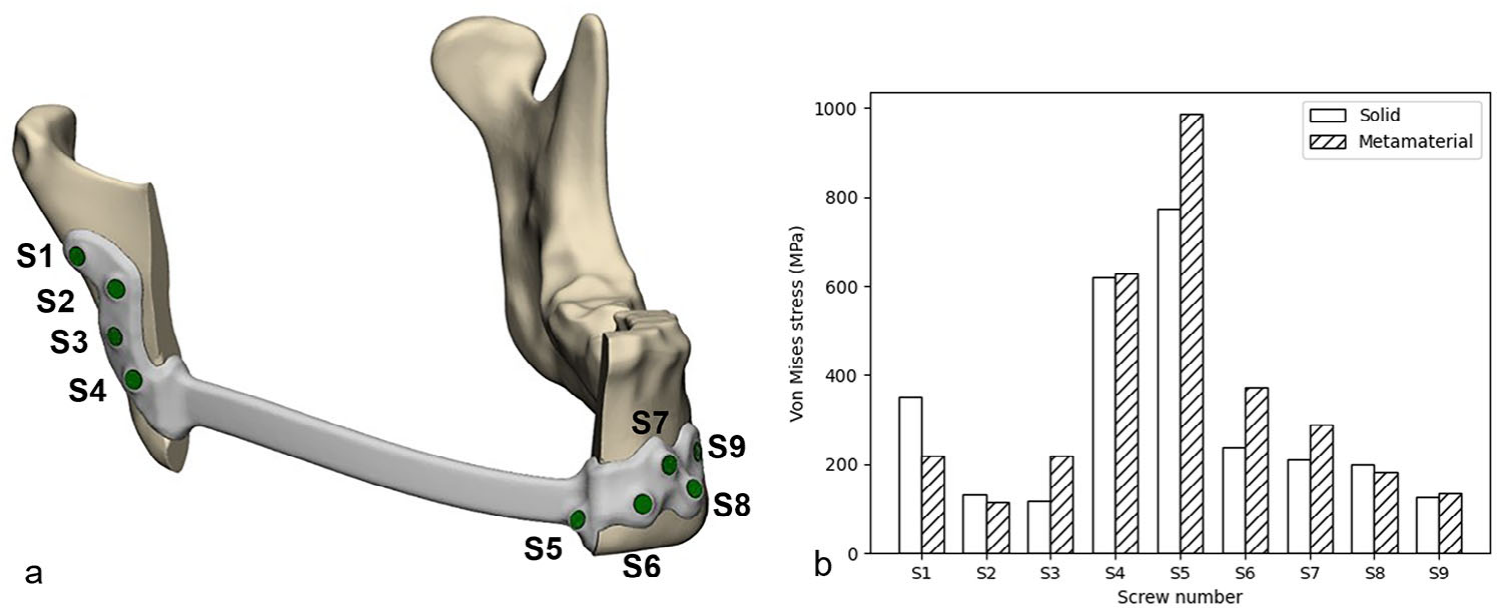
Screw (S) numbering a) and the maximum values of the Von Mises stresses on the screws b) from the patient‐specific solid and metamaterial plate load reconstruction tests.

We first focused on estimating the maximal stress induced by the screws (**Figure** **9**a) as it contributes to stress shielding. For this, the maximal Von Mises stress values were extracted per screw under a force load of 1 kN. The solid and metamaterial reconstruction plate results shown in 9b reveal that the metamaterial plate's screws close to the defect (screws 3–7) experienced higher stresses compared to the solid plate's screws. On the other hand, the solid reconstruction plate's screws located further away from the defect (screw 1, 2, 8) were characterized by less stress. Screw 9 was, however, an exception: the stresses observed there, 128 and 134.1 MPa for the solid plate and the metamaterial plate, respectively, differed only marginally.

**Figure 10:**
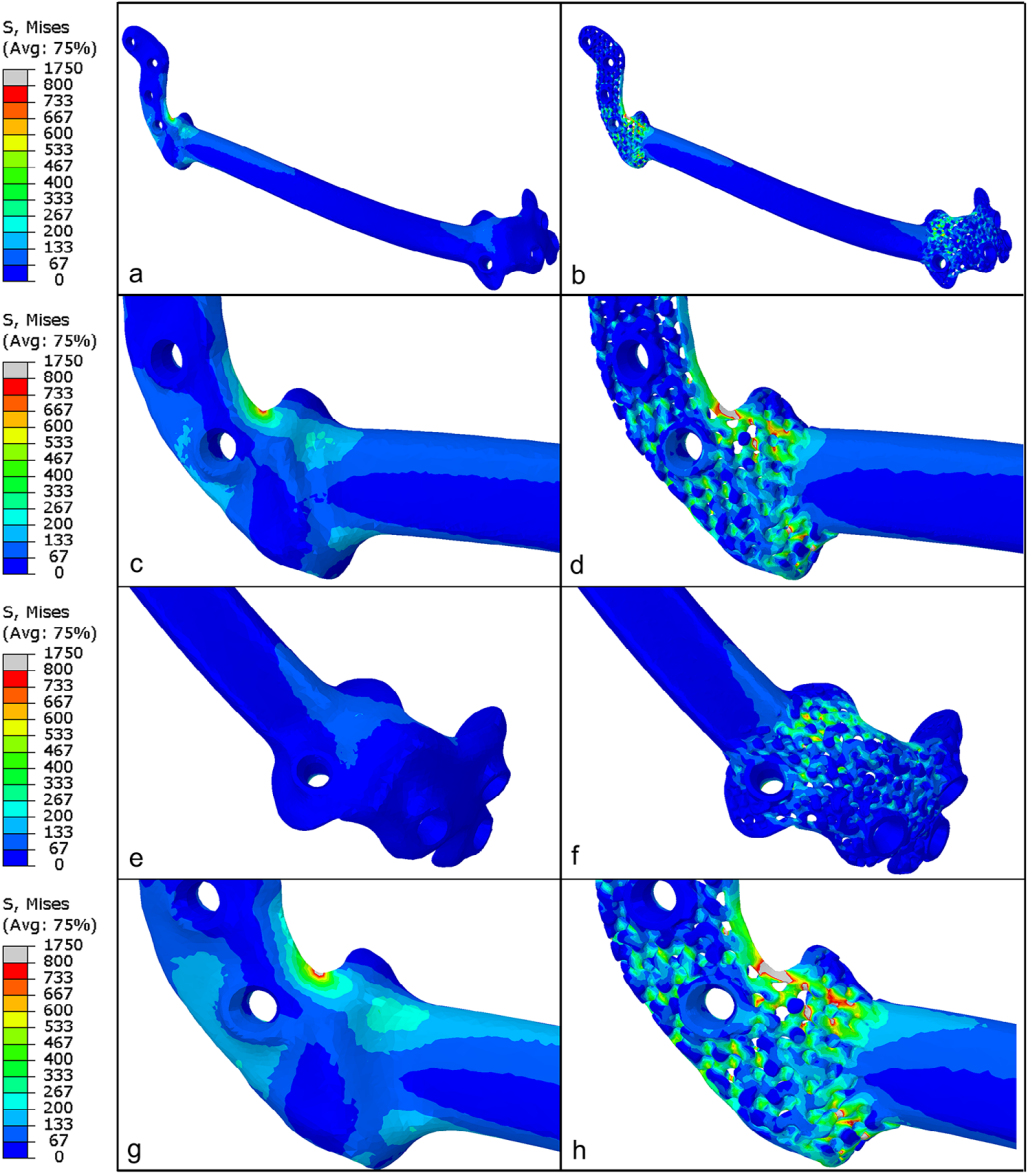
Stress fields (S) in the solid and metamaterial reconstruction plates. a and b are the overall stress distribution of the solid and metamaterial reconstruction plates, respectively, under a compression load of 500 N. c, d and e, f are the zoomed‐in‐views of right and left flanges of the solid and metamaterial reconstruction plates, respectively, under a 500 N load. g and h are the zoomed‐in‐views of the solid and metamaterial reconstruction plates, respectively, under a 600 N load.

The finite‐element analysis enabled an estimation of the stresses within the reconstruction plates to give useful insights into the mechanical behavior of the proposed designs and failure mode predictions. The stress fields of the solid and metamaterial plates loaded with 500 N (**Figure** **10**a–f) had similar stress distribution patterns, with stress concentrations near the sharp edge in the right segment of the plates. Note that the colors in the figures ranged from 0 to 800 MPa, where the upper limit was equal to the yield strength of the titanium alloy.^[^ ^38^ ^]^ The metamaterial reconstruction plate clearly exhibited higher stresses in the thinnest parts of the microstructure, some of which even exceeded 800 MPa. However, no failure was expected under the applied load since none of the microstructural elements were completely grey. On increasing the load to 600 N, some struts greyed out completely, indicating failure, as can be seen in Figure 10g,h. This was also confirmed by our observations during the experiments described above.

**Figure 11:**
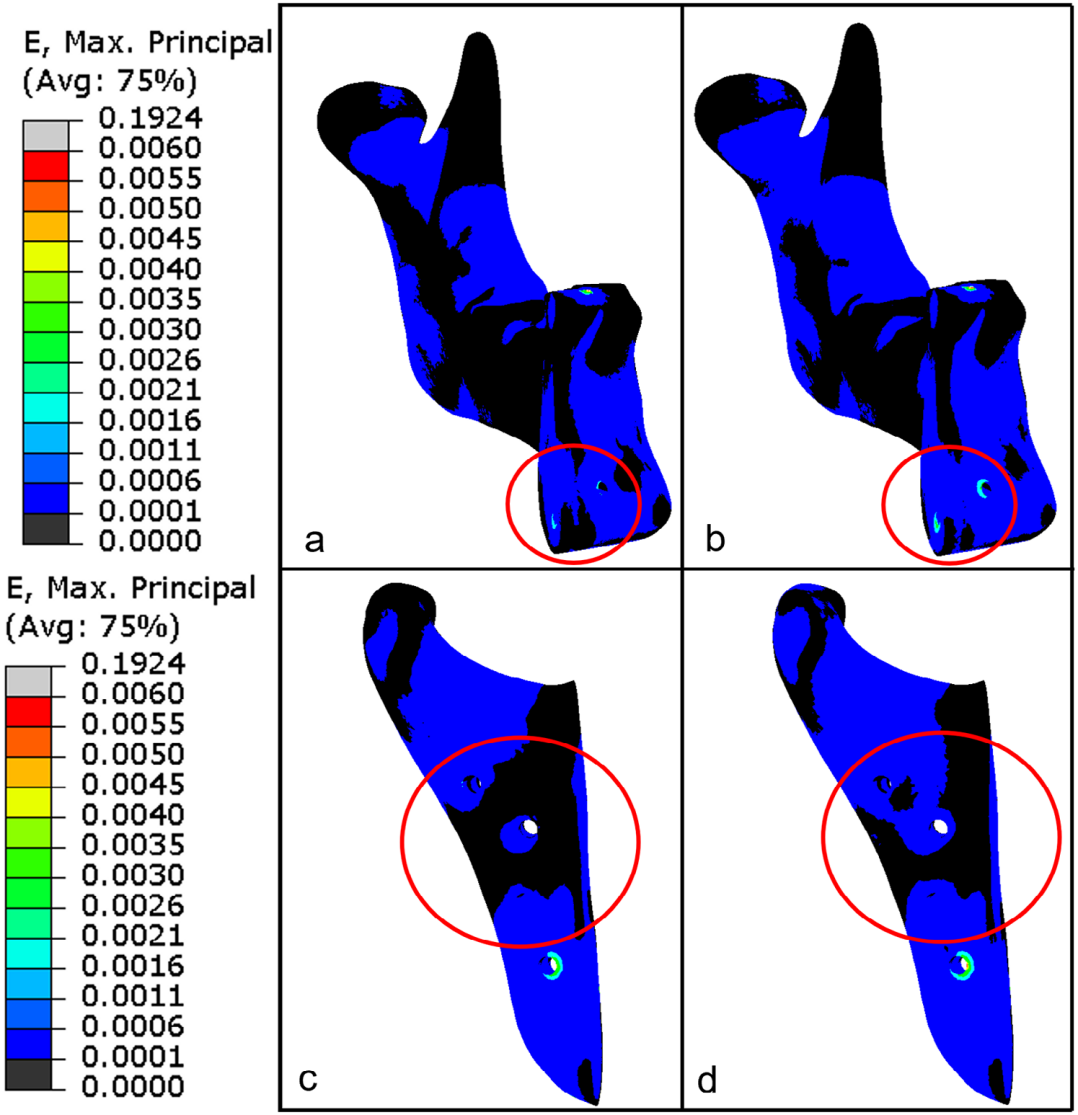
Strain (E) fields in the mandible segments under a 200 N load: the left mandible segment bridged by the (a) solid and (b) metamaterial reconstruction plates, and the right mandible segment bridged by the (c) solid and (d) metamaterial reconstruction plates.

Next, we visualize the strain distribution of a mandible loaded with 200 N (**Figure** **11**). It can be seen that the bone segments assembled with the metamaterial reconstruction plate experienced more strain in the regions where stress‐shielding is usually observed (encircled by red). Note that the leading colors correspond to the strains, ranging from 0.0001 [‐] to 0.006 [‐], under which bone does not resorb due to under‐ or overloading.^[^ ^39^, ^40^ ^]^

The stress fields in the mandible segments were studied in two separate numerical studies, with the Clear V4 Resin and bone properties for the bone segments, respectively. In the latter case, the bones were represented by 2 mm thick shells representing the cortical bone. The maximum values of the Von Misses stresses for areas 1–6 (indicated in Figures 5, 6, 7, 8) are given in **Table** **2**, together with the isochromatic fringe counts for both the solid and the metamaterial reconstruction plates. The magnitudes of the stresses obviously differed due to different mechanical properties; the qualitative trends of their distribution per area were, however, very similar, confirming the validity of undertaking photoelastic tests to study stress shielding.

**Table 2:** Isochromatic fringe counts, and Von Mises stress from finite element (FEA) analysis of the Clear V4 Resin mandible segments and of the cortical bone mandible segments of the solid plate and the metamaterial plate, per area indicated in Figures 5, 6, 7, 8.

Area Number	Isochromatic fringe count		FEA Von Mises stress [Clear V4 Resin] [MPa]		FEA Von Mises stresses [cortical bone] [MPa]	
	Solid plate	Metamaterial plate	Solid plate	Metamaterial plate	Solid plate	Metamaterial plate
1	6	5	2.4	2.3	7.5	7.9
2	7	5	8.6	7.8	13.6	12.8
3	5	4	6	5.7	17.1	16.6
4	5	3	10.8	11.4	19	19.9
5	5	8	2.5	3.9	10.5	13.3
6	5	7	1.6	2.3	5.7	8.4

### 3.3 Statistical Analysis

To assess the quantitative agreement between the isochromatic fringe count and the finite‐element estimations of the Von Mises stresses, we performed a Pearson correlation analysis, and the results indicated a significant positive correlation between the experimental fringes and Von Mises stresses, with r = 0.776 and p = 0.003. This means that as the isochromatic fringe count increased, the numerical value of the Von Mises stress also increased. The correlation is significant at the 0.01 level (2‐tailed), indicating a strong relationship between the two variables.

## 4 Discussion

We present the first visual and quantitative estimations of stress shielding induced by conventional, patient‐specific and metamaterial mandibular reconstruction plates by combining photoelastic experiments and finite‐element studies.

Our results reveal substantial variations in the stress distributions in the mandible on testing two different plate designs, confirming the initial hypothesis that the metamaterial reconstruction plate can increase stresses and thus reduce stress shielding if properly designed. Even though this study only tested a homogeneous, non‐optimal metamaterial with a periodic micro‐structure, it shows several advantages over a fully solid material. For instance, the metamaterial parts of the reconstruction plate increase the local stresses around the screws in proximity to the mandibular defect. Since the effective stiffness of the metamaterial plate is lower than that of the solid plate, the metamaterial acts as a softer medium than the screws. This results in a load redistribution, i.e., more load is transmitted in the areas near the mandible defect and less load reaches the screws which are further from the defect. This is clearly seen by examining the isochromatic fringe counts in Figures 5, 6, 7, 8. Since stress shielding mainly occurs in the bone areas near defects due to the lack of mechanical stimuli, increasing the stresses is considered favorable for reducing stress shielding.^[^ ^41^ ^]^ Furthermore, increasing these local stresses is crucial for maintaining stable bone density and avoiding bone resorption, which are closely related to implant failure.^[^ ^6^, ^40^ ^]^

Yet, the metamaterial reconstruction plate induces lower stress in the remaining mandible segments located further away from the defect. This is underscored by the higher isochromatic fringe counts for the solid implant screws compared to those for the metamaterial implant (Figures 5, 6, 7, 8). Due to the relatively lower stiffness, the metamaterial plate bends and deforms more compliantly compared to its solid counterpart. Therefore, more energy is stored in the form of potential energy within the metamaterial plate that reduces the stresses transferred to the mandible parts located further from the defect. The energy distribution can, however, be controlled effectively by optimizing the design and dimensions of the metamaterial parts, but this is beyond the scope of our study.

Our finite‐element results agree very well with the experimental observations and, additionally, provide quantitative data about the stress‐strain fields in different parts of the analyzed models. The magnitude of the estimated maximum values of the Von Mises stress for the screws, the plates and the mandible segments are similar to those observed experimentally, confirmed by the strong positive correlation in the statistical analysis. This strengthens the statement that metamaterials can be used successfully to reduce stress shielding induced by metal reconstruction plates.

The structural performance of the loaded samples presents a clear trade‐off between the mechanical strength of a reconstruction plate and its biomechanical optimization. The metamaterial plate failed under 775 N, after local fractures had occurred, as revealed by clear audible snapping sounds at 600 and 700 N, indicating progressive micro‐structural failures. This gradual failure process suggests that certain elements within the gyroid microstructure can bear higher loads than others, which is confirmed by the numerically estimated stress fields shown in Figure 10. To increase the survival rate of the metamaterial plate, its design could be optimized further by mitigating stress concentrations due to more rounded edges and fillets, or by thickening the elements experiencing high stress. Moreover, the current design could be enhanced by surface hardening treatments.^[^ ^42^ ^]^ In contrast, the solid reconstruction plate withstood loads of up to 1800N without failure. This difference in the failure resistance shows the robustness of the personalized solid plate. However, such mechanical robustness comes at the cost of biomechanical functioning in terms of the associated stress shielding.

We also need to mention the limitations of our study. Despite the similarity of the stress patterns in the transparent resin mandible models with those in cortical bone, the mechanical behavior of the resin differs substantially from that of bone, in terms of structure, anisotropy, fracture, porosity, and viscosity. Thus, the experimental results can only be used to describe the behavior of a real mandible in a very approximate sense. Next, we only studied one sample of each implant due to high manufacturing costs and long production times. This makes it impossible to study the variations in and effects of geometric features of patient‐specific designs of mandibular reconstruction plates. Moreover, the finite‐element studies were limited to static loading conditions, and did not account for cyclic loading or dynamically induced fatigue, which are critical in the mastication context. Further research will be conducted to estimate the fatigue behavior of mandible reconstruction plates based on dynamic loading conditions.^[^ ^12^, ^43^ ^]^ Finally, numerous other metamaterial part designs can deliver better mechanical performance in terms of reducing stress shielding;^[^ ^16b^ ^]^ the designs could be optimized to account for patient‐specific morphology and situation. In this sense, metamaterials can, theoretically, open up unlimited design freedom to develop truly stress‐shielding‐free implants.

Overall, our results confirm the expected potential of metamaterials to address the issues related to stress shielding that can potentially prevent bone resorption and screw loosening, followed by reconstruction failure thus, ultimately, improving conditions for patients. The enhanced mechanical stimulus provided by the metamaterial plate is particularly beneficial for preserving bone integrity in the critical regions near bone defects. However, the reduced load‐bearing capacity of the metamaterial mandible reconstruction plate may introduce certain limitations to its clinical application if the metamaterial's design is not optimized to a patient's conditions. Besides, before being put into clinical use, the plate must be proven to withstand cyclic loads introduced by biting and chewing for the rest of the patient's life. Yet, one needs to make a trade‐off between reduced stress shielding and the potential risk of an implant failure. Given the nature of a mandible after a resection and reconstruction, one would expect masticatory forces to be considerably lower postoperatively compared to preoperatively. These reduced functional demands can be beneficial for the survivability of metamaterial implants.

## 5 Conclusion

This study demonstrates that metamaterial‐based mandibular implants redistribute stresses effectively, and enhance mechanical stimulation in critical bone, thereby reducing stress shielding. Based on photoelasticity experiments and numerical FEA, it was found that incorporating a gyroid metamaterial structure improves load transfer. Specifically, the metamaterial plate increases stresses in critical regions near the mandibular defect. This was highlighted by the isochromatic fringe counts of 8 and 7 for the metamaterial plate versus the counts of 5 and 5 for the solid plate. The stress‐values found during finite element analysis (P = 0.003) validated the findings further. Such improved load transfer enhances mechanical stimulation, reducing bone loss in areas prone to bone resorption. The mechanical strength of the metamaterial plate was lower than that of the solid plate, with ultimate failure occurring at a load of 775 N. The solid plate was loaded up to 1800 N without signs of failure. This highlights the trade‐off between reducing stress‐shielding while maintaining load‐bearing capacity. Our results emphasize the need for further research focusing on optimizing metamaterial properties, investigating hybrid designs, and evaluating dynamic loading and fatigue behavior to advance the clinical applicability of these implants. The study highlights the clinical potential of metamaterial implants in reducing bone resorption, screw loosening, and implant failure, ultimately enhancing patient outcomes. Further research will be conducted on the magnitude of masticatory forces that occur postoperatively, and how the metamaterial reconstruction plates can be optimally altered. Further research will include exploring other relative densities, alternative unit cell designs, or hybrid plates integrating both solid and metamaterial elements. All‐in‐all, this study highlights the potential of metamaterial designs to reduce stress shielding and preserve bone integrity, offering a promising direction for patient‐specific mandible reconstruction plates.

## Conflict of Interest

The authors declare no conflict of interest.

## Data Availability

Research data are not shared.

## References

[1] A. P. Singh , M. Rana , B. Pal , P. Datta , S. Majumder , A. Roychowdhury , Med. Eng. Phys. 2023, 113, 103959. 36965999 10.1016/j.medengphy.2023.103959

[2] H. Mehboob , Compos. Struct. 2023, 303, 116379.

[3] A. van Kootwijk , V. Moosabeiki , M. C. Saldivar , H. Pahlavani , M. A. Leeflang , S. Kazemivand Niar , P. Pellikaan , B. P. Jonker , S. M. Ahmadi , E. B. Wolvius , N. Tumer , M. J. Mirzaali , J. Zhou , A. A. Zadpoor , J. Mech. Behav. Biomed. Mater. 2022, 132, 105291. 35660552 10.1016/j.jmbbm.2022.105291

[4] A. Ouldyerou , A. Merdji , L. Aminallah , S. Roy , H. Mehboob , M. Özcan , J. Mech. Behav. Biomed. Mater. 2022, 134, 105422. 36037710 10.1016/j.jmbbm.2022.105422

[5] R. Huiskes , J. D. Janssen , T. J. Slooff , In Mechanical Properties of Bone (Ed.: S. C. Cowing ), The American Societey of Mechanical Engineers, New York 1981, pp. 211–234.

[6] a) R. Allena , D. Scerrato , A. Bersani , I. Giorgio , Mech. Res. Commun. 2023, 129, 104094;

[7] W. N. Andrade , J. E. Lipa , C. B. Novak , H. Grover , C. Bang , R. W. Gilbert , P. C. Neligan , Head Neck 2008, 30, 341. 17902165 10.1002/hed.20705

[8] S. Elsayed , Y. Ahmed , M. I. El‐Anwar , E. Elddamony , R. Ashraf , BMC Oral Health 2025, 25, 166. 39885486 10.1186/s12903-025-05440-5 PMC11783779

[9] a) S. Arabnejad , B. Johnston , M. Tanzer , D. Pasini , J. Orthop. Res. 2017, 35, 1774; 27664796 10.1002/jor.23445

[10] a) C. A. Engh, Jr. , A. M. Young , C. A. Engh, Sr. , R. H. Hopper, Jr. , Clin. Orthop. Relat. Res. 2003, 417, 157; 10.1097/01.blo.0000096825.67494.e3 14646713

[11] a) J. J. Lang , M. Bastian , P. Foehr , M. Seebach , J. Weitz , C. von Deimling , B. J. Schwaiger , C. M. Micheler , N. J. Wilhelm , C. U. Grosse , PLoS One 2021, 16, 0253002; 10.1371/journal.pone.0253002 PMC8186800 34101755

[12] a) B. B. J. Merema , F. K. L. Spijkervet , J. Kraeima , M. J. H. Witjes , Sci. Rep. 2025, 15, 644; 39753636 10.1038/s41598-024-82964-w PMC11698898

[13] a) R. H. Khonsari , P. Berthier , T. Rouillon , J.‐P. Perrin , P. Corre , J. Oral Maxillofac. Surg., Med. Pathol. 2014, 26, 477;

[14] D. E. Las , D. Verwilghen , M. Y. Mommaerts , J. Cranio‐Maxillofac. Surg. 2021, 49, 34. 10.1016/j.jcms.2020.10.002 33257187

[15] a) H. M. Kolken , S. Janbaz , S. M. Leeflang , K. Lietaert , H. H. Weinans , A. A. Zadpoor , Mater. Horiz. 2018, 5, 28;

[16] a) A. O. Krushynska , D. Torrent , A. M. Aragón , R. Ardito , O. R. Bilal , B. Bonello , F. Bosia , Y. Chen , J. Christensen , A. Colombi , Nanophotonics 2023, 12, 659; 39679340 10.1515/nanoph-2022-0671 PMC11636487

[17] L. Zhang , B. Song , S.‐K. Choi , Y. Shi , Int. J. Mech. Sci. 2021, 197, 106331.

[18] S. A. Naghavi , M. Tamaddon , P. Garcia‐Souto , M. Moazen , S. Taylor , J. Hua , C. Liu , Front. Bioeng. Biotechnol. 2023, 11, 1092361. 36777247 10.3389/fbioe.2023.1092361 PMC9910359

[19] a) S. M. Ghalehney , M. H. Sadeghi , H. Barati , H. Gharehbaghi , Phys. Scr. 2024, 99, 115941;

[20] K. M. Hijazi , S. J. Dixon , J. E. Armstrong , A. S. Rizkalla , Materials (Basel) 2023, 17, 140. 38203994 10.3390/ma17010140 PMC10779528

[21] S. Kumar , S. Tan , L. Zheng , D. M. Kochmann , npj Comput. Mater. 2020, 6, 73.

[22] K. Barri , Q. Zhang , I. Swink , Y. Aucie , K. Holmberg , R. Sauber , D. T. Altman , B. C. Cheng , Z. L. Wang , A. H. Alavi , Adv. Funct. Mater. 2022, 32, 2203533.

[23] J. Luo , W. Lu , P. Jiao , D. Jang , K. Barri , J. Wang , W. Meng , R. P. Kumar , N. Agarwal , D. K. Hamilton , Mater. Today 2025, 83, 145.

[24] P. Jiao , C. Zhang , W. Meng , J. Wang , D. Jang , Z. Wu , N. Agarwal , A. H. Alavi , ACS Appl. Mater. Interfaces 2025, 17, 2991. 39746033 10.1021/acsami.4c17625 PMC11744508

[25] I. Giorgio , M. Spagnuolo , U. Andreaus , D. Scerrato , A. M. Bersani , Mathemat. Mech. Solids 2021, 26, 1074.

[26] D. B. Alemayehu , M. Todoh , S.‐J. Huang , J. Funct. Biomater. 2025, 16, 54. 39997588 10.3390/jfb16020054 PMC11856169

[27] a) M. Cehreli , J. Duyck , M. De Cooman , R. Puers , I. Naert , Clin. Oral Implants Res. 2004, 15, 249; 15008938 10.1111/j.1600-0501.2004.00979.x

[28] B. P. Kumar , V. Venkatesh , K. A. Kumar , B. Y. Yadav , S. R. Mohan , J Maxillofac Oral Surg 2016, 15, 425. 27833334 10.1007/s12663-015-0766-5 PMC5083680

[29] a) A. Askari , M. Jamalzadeh , AIP Adv. 2024, 14, 100702;

[30] a) A. P. G. Castro , R. B. Ruben , S. B. Goncalves , J. Pinheiro , J. M. Guedes , P. R. Fernandes , Comput. Methods Biomech. Biomed. Eng. 2019, 22, 567; 10.1080/10255842.2019.1569638 30773050

[31] a) D. M. Kochmann , J. B. Hopkins , L. Valdevit , Mrs Bulletin 2019, 44, 773;

[32] J. Y. Rho , R. B. Ashman , C. H. Turner , J. Biomech. 1993, 26, 111. 8429054 10.1016/0021-9290(93)90042-d

[33] a) C. Hou , M. Goris , D. Rosseel , B. Vrancken , K. Denis , J. Manufactur. Mater. Process. 2024, 8, 256;

[34] a) C. Herráez‐Galindo , D. Torres‐Lagares , Á.‐J. Martínez‐González , A. Pérez‐Velasco , E. Torres‐Carranza , M.‐A. Serrera‐Figallo , J.‐L. Gutiérrez‐Pérez , Metals 2020, 10, 648;

[35] a) D. C. Koper , C. A. W. Leung , L. C. P. Smeets , P. F. J. Laeven , G. J. M. Tuijthof , P. Kessler , J. Mech. Behav. Biomed. Mater. 2021, 113, 104157; 33187871 10.1016/j.jmbbm.2020.104157

[36] a) P. Takaki , M. Vieira , S. Bommarito , Int. Arch. Otorhinolaryngol. 2014, 18, 272; 25992105 10.1055/s-0034-1374647 PMC4297017

[37] D. Farnsworth , P. E. Rossouw , R. F. Ceen , P. H. Buschang , Am. J. Orthod. Dentofacial Orthop. 2011, 139, 495. 21457860 10.1016/j.ajodo.2009.03.057

[38] K. Moiduddin , J. Med. Biolog. Eng. 2018, 38, 744.

[39] S. Buvinic , J. Balanta‐Melo , K. Kupczik , W. Vasquez , C. Beato , V. Toro‐Ibacache , Front. Endocrinol. (Lausanne) 2020, 11, 606947. 33732211 10.3389/fendo.2020.606947 PMC7959242

[40] H. M. Frost , Angle Orthod 1994, 64, 175. 8060014 10.1043/0003-3219(1994)064<0175:WLABSA>2.0.CO;2

[41] a) K. Darwich , M. B. Ismail , M. Y. A. Al‐Mozaiek , A. Alhelwani , Oral. Maxillofac. Surg. 2021, 25, 103; 32725572 10.1007/s10006-020-00889-w

[42] C. N. Kelly , N. T. Evans , C. W. Irvin , S. C. Chapman , K. Gall , D. L. Safranski , Mater. Sci. Eng. C Mater. Biol. Appl. 2019, 98, 726. 30813077 10.1016/j.msec.2019.01.024 PMC6400308

[43] I. Giorgio , U. Andreaus , D. Scerrato , P. Braidotti , Mathemat. Mech. Solids 2017, 22, 1790.

